# Transcatheter Arterial Embolization for Bleeding From the Proper Hepatic Artery Caused by a Duodenal Ulcer: A Case Report

**DOI:** 10.7759/cureus.63822

**Published:** 2024-07-04

**Authors:** Masaki Imaeda, Yasuyuki Onishi, Shu Nagatomo, Ryuki Minami, Takanori Taniguchi

**Affiliations:** 1 Department of Radiology, Tenri Hospital, Tenri, JPN; 2 Department of Diagnostic Imaging and Nuclear Medicine, Kyoto University, Kyoto, JPN; 3 Department of Gastroenterology, Tenri Hospital, Tenri, JPN

**Keywords:** computed tomography, transcatheter arterial embolization, proper hepatic artery, duodenal ulcer, bleeding

## Abstract

Although bleeding is the most common complication of peptic ulcer disease, bleeding from the proper hepatic artery is unusual. We report on the case of an 87-year-old woman who presented with melena. An upper endoscopy was performed for a bleeding duodenal ulcer; however, the bleeding could not be controlled. A careful assessment using contrast-enhanced computed tomography (CT) demonstrated that the bleeding source was the proper hepatic artery. Transcatheter arterial embolization of the proper hepatic artery was successfully performed. This case highlights the importance of careful assessment using contrast-enhanced CT to identify the source of bleeding. Endovascular treatment is the first choice of treatment for patients with bleeding from large arteries.

## Introduction

Gastric and duodenal ulcers together are called peptic ulcer diseases, with an estimated lifetime prevalence of 5-10% and an annual incidence of 0.1-0.3% [1]. Bleeding is the most frequent complication of peptic ulcer disease and occurs in approximately 15-20% of patients [2]. Endoscopy is recommended for the diagnosis and treatment of peptic ulcer disease, whereas transcatheter arterial embolization (TAE) is recommended when endoscopy is not feasible [3]. In cases of TAE, the most common causative artery is the gastroduodenal artery [4]. Hemorrhage of the proper hepatic artery is rare. In previous case reports, bleeding from the proper hepatic artery was not controlled by endoscopy, and TAE or surgical treatment was required [5-7]. Herein, we report a case of proper hepatic artery bleeding caused by a duodenal ulcer that was successfully treated with TAE.

## Case presentation

An 87-year-old woman called an ambulance because of melena. She had been prescribed oral non-steroidal anti-inflammatory drugs (NSAIDs) for one month after a lumbar compression fracture from a fall. Notably, she had experienced an episode of melena two weeks prior, although she had not sought medical attention at that time. She had a history of untreated type 2 diabetes, hypertension, and osteoporosis, but no history of surgery. At the time of emergency medical service contact, her level of consciousness was clear, and her vital signs were within normal limits. Immediately after arrival at the hospital, she vomited a large amount of blood, and her systolic blood pressure decreased to 80 mmHg. Her vital signs recovered in response to a 1 L fluid infusion. Blood tests revealed anemia and elevated levels of inflammatory markers (Table 1).

**Table 1:** Blood test results. AST: aspartate transaminase; ALT: alanine transaminase; LDH: lactate dehydrogenase; ALP: alkaline phosphatase; CRP: C-reactive protein

Test	Result	Reference range
White blood cells	17140	3.3–8.6 × 10^3^/uL
Hemoglobin	6.5	11.6–14.8 g/dL
Platelets	351000	158–348 × 10^3^/uL
AST	55	13–30 U/L
ALT	126	7–23 U/L
LDH	164	124–222 U/L
ALP	413	38–113 U/L
Total bilirubin	0.5	0.4–1.5 mg/dL
CRP	8.13	≤0.14 mg/dL

**Figure 1:**
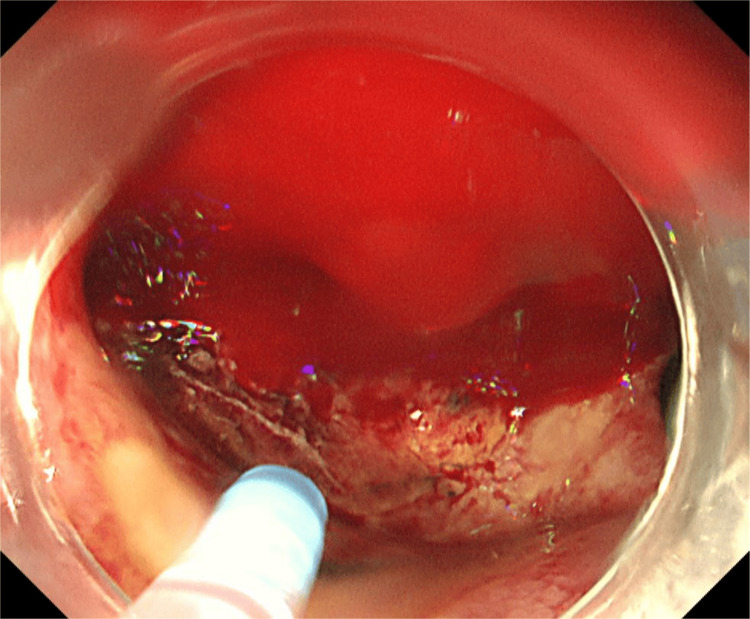
Endoscopic image showing a deep ulcer in the duodenal bulb with active bleeding.

Contrast-enhanced computed tomography (CT) showed an ulcer in the duodenal bulb, which was considered the source of bleeding. An emergency upper endoscopy revealed a deep ulcer that was actively spurting blood in the duodenal bulb (Figure 1).

**Figure 2:**
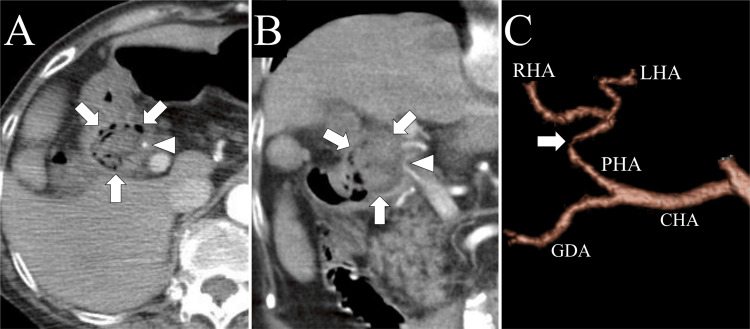
Contrast-enhanced computed tomography (CT) at presentation. Axial (A) and coronal (B) arterial-phase contrast-enhanced CT images show a duodenal ulcer (arrows) at the duodenal bulb. The proper hepatic artery (arrowheads) is observed at the base of the ulcer. (C) 3D reconstruction of the hepatic artery from arterial-phase CT showing severe stenosis (arrow) in the proper hepatic artery. The site of stenosis was located at the base of the duodenal ulcer. CHA: common hepatic artery; GDA: gastroduodenal artery; LHA: left hepatic artery; PHA: proper hepatic artery; RHA: right hepatic artery

Despite using high-frequency forceps and hemostatic clips, complete hemostasis was not achieved because of intense bleeding. Therefore, we decided to perform a TAE to stop the bleeding. Six units of red blood cells were transfused during the endoscopy. A meticulous assessment of contrast-enhanced CT before TAE revealed that the proper hepatic artery ran along the bottom of the duodenal ulcer and narrowed at the bottom of the ulcer (Figures 2A, 2B), indicating bleeding from the proper hepatic artery. A 3D reconstruction of the hepatic artery using arterial-phase CT data demonstrates a severe stenosis (arrow) in the proper hepatic artery (Figure 2C). The site of stenosis was located at the base of the duodenal ulcer.

**Figure 3:**
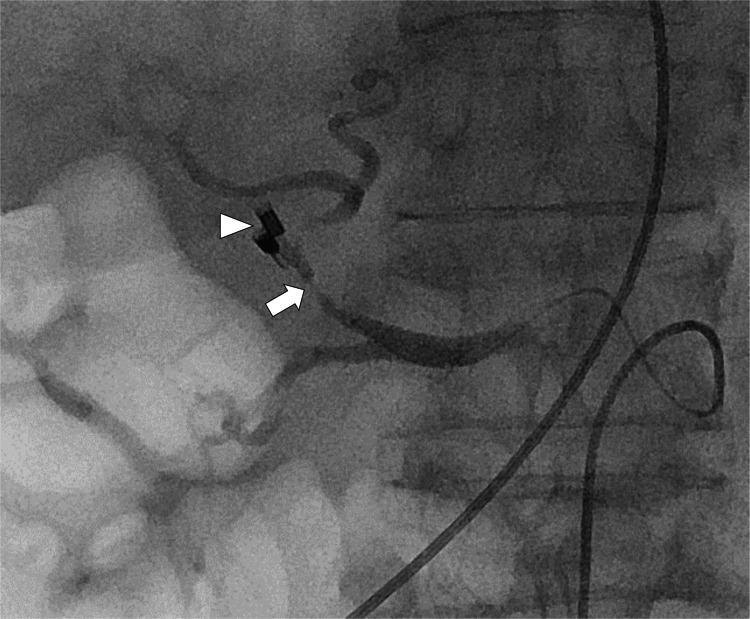
Angiography of the proper hepatic artery before embolization showing severe stenosis in the proper hepatic artery (arrow). The stenosis was located close to the hemostatic clips (arrowhead) placed during the endoscopy.

At the start of the embolization procedure, the patient was restless and hypotensive, and the procedure was performed under local anesthesia with a rapid transfusion of red blood cells. The right femoral artery was punctured, and a 4-F sheath was placed. A 4-F curved catheter (Rosch Celiac) was advanced through the sheath to catheterize the celiac artery. Angiography of the proper hepatic artery conducted using a microcatheter revealed severe stenosis in the proper hepatic artery, and the clips used for upper endoscopy were located close to the stenosis (Figure 3).

**Figure 4:**
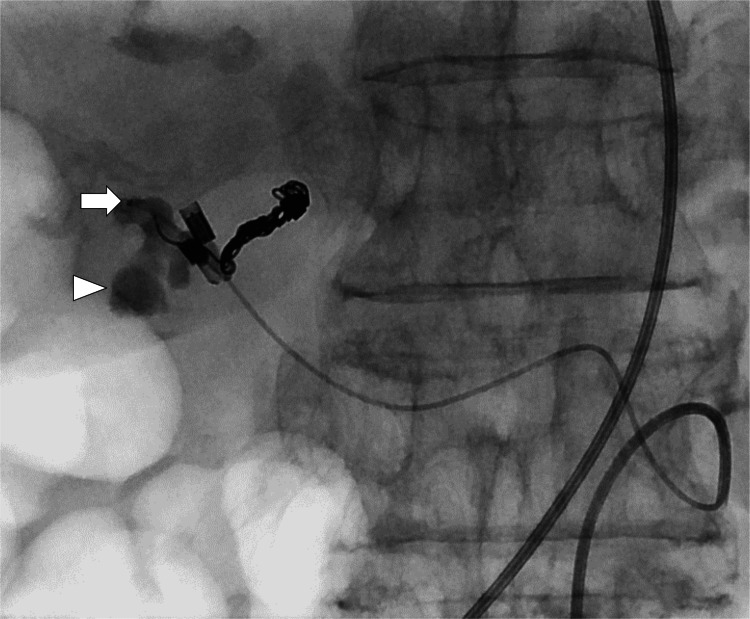
Fluoroscopic image during embolization showing the tip of the microcatheter (arrow) in the duodenal lumen. Contrast medium injected from the microcatheter was observed in the duodenum (arrowhead).

No extravasation of contrast medium or pseudoaneurysms was observed. Subsequently, the proper hepatic artery was embolized using microcoils. During embolization, the microcatheter was unintentionally advanced outside the hepatic artery near the clips (Figure 4).

**Figure 5:**
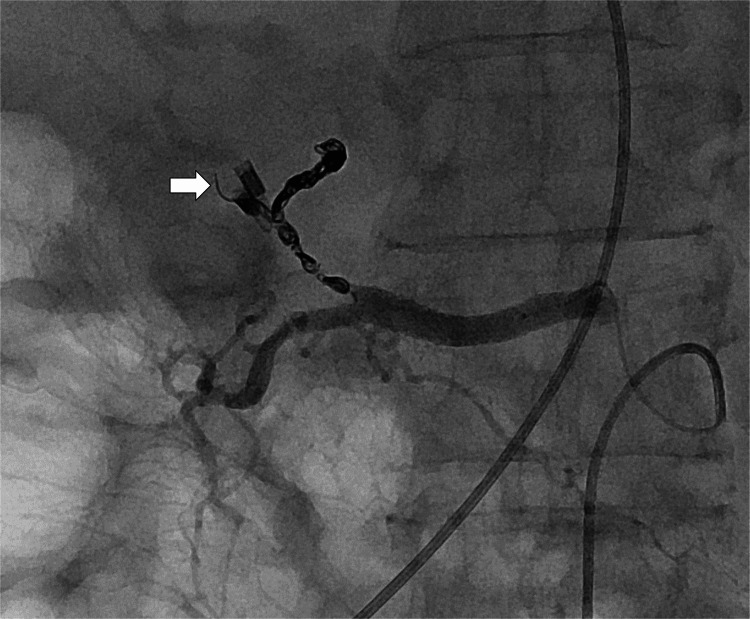
Angiography of the common hepatic artery after coil embolization of the proper hepatic artery showing successful occlusion of the proper hepatic artery. Protrusion of a coil end (arrow) into the duodenal ulcer is observed.

Microcatheter angiography revealed that the catheter tip was located in the duodenum. These findings confirmed that the proper hepatic artery was the site of bleeding. Angiography of the common hepatic artery after embolization revealed successful hemostasis of the proper hepatic artery (Figure 5).

**Figure 6:**
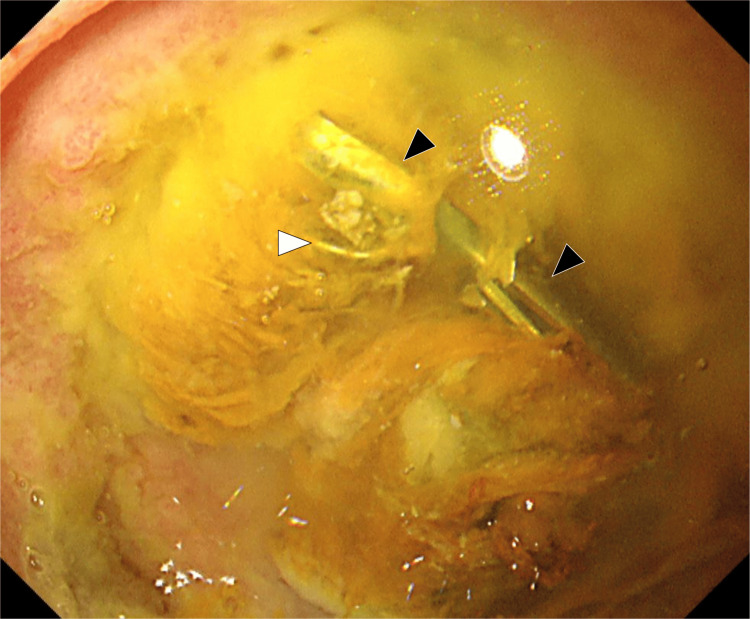
Endoscopic image after embolization showing a healing duodenal ulcer. A coil (white arrowhead) and hemostatic clips (black arrowheads) are observed.

After the procedure, the patient's restlessness improved, consciousness became clear, and vital signs stabilized. Oral intake was initiated the following day, and the patient did not experience recurrent hematemesis or abdominal pain. Blood tests conducted on the same day revealed elevated liver enzyme levels: aspartate transaminase/alanine transaminase (AST/ALT): 220/117 IU/L. However, blood tests conducted one week later demonstrated normalization of liver enzyme levels and no progression of anemia. One week after TAE, contrast-enhanced CT showed no signs of hepatic infarction, and upper endoscopy revealed an ulcer on the anterior wall of the duodenal bulb, categorized as stage A2, with signs of healing (Figure 6).

On the 22nd day after TAE, the patient was transferred to another hospital without any complications.

## Discussion

The proper hepatic artery is a rare site of arterial bleeding caused by peptic ulcers. Only a few cases of proper hepatic artery bleeding caused by peptic ulcers have been reported [5-7]. The majority of the reported cases involved bleeding at the anastomotic site of the hepatic artery after liver transplantation with a right lobe liver graft [7,8]. The possible mechanisms of proper hepatic artery bleeding in recipients of right lobe liver grafts include dissection around the porta hepatis, anatomical proximity of the hepatic artery anastomosis to the duodenal bulb, and exposure of the arterial anastomosis to duodenal fluid contained by the post-operative adhesions [7]. In contrast, the present patient had no history of abdominal surgery, which is unusual. In the present case, long-term use of NSAIDs was thought to have caused a duodenal ulcer. In all the previous cases, bleeding from the hepatic artery was not controlled by endoscopy alone and required TAE and/or surgical intervention. Endovascular treatment is more suitable than endoscopic hemostasis for hepatic arterial bleeding caused by duodenal ulcers. In patients with bleeding peptic ulcer disease, contrast-enhanced CT images should be carefully evaluated to identify the vessels that cause bleeding. If bleeding from a large artery, such as the proper hepatic artery, is suspected, we suggest endovascular treatment instead of endoscopy.

Proper hepatic artery bleeding can be treated with TAE or stent-graft placement. The Society for Vascular Surgery guidelines recommend stent-graft placement for hepatic artery aneurysms when feasible and recommend resection of the aneurysms with interposition grafts when stent-graft placement is not feasible [9]. The guidelines do not recommend TAE for hepatic artery aneurysms due to the risk of hepatic failure. However, TAE of the hepatic artery does not always result in ischemic liver damage [10,11]. Portal vein stenosis and coagulopathy have been reported as risk factors for liver failure [11,12]. The present patient did not have these two risk factors, and the risk of ischemic liver damage was not high. Stent grafts were unavailable at the time of the procedure. Therefore, TAE of the proper hepatic artery was an acceptable treatment option.

## Conclusions

Here, we report a case of proper hepatic artery bleeding caused by a duodenal ulcer. The bleeding was not controlled by endoscopy, and TAE was performed for hemostasis. In patients with bleeding peptic ulcer disease, it is important to carefully read contrast-enhanced CT scans to identify the source of bleeding. Endovascular treatment, rather than endoscopy, should be the first choice of treatment for bleeding from large arteries.
